# Supplementary material for: Metagenome-wide association of gut microbiome features for schizophrenia
Source: Nat Commun. 2020 Mar 31;11:1612. doi: 10.1038/s41467-020-15457-9 (PMC7109134; doi:10.1038/s41467-020-15457-9)
Supplement: Supplementary file 1 — Supplementary Information [file 41467_2020_15457_MOESM1_ESM.docx]

**Metagenomic profiling of the gut microbiota in schizophrenia**

Zhu et al.

**Supplementary information**

**Online methods**

**Supplementary Figure 1~9**

**Online Methods**

**Subject recruitment**

All subjects participated voluntarily and signed a written informed consent prior to their enrollment. The case group only included acutely relapsed schizophrenic (ARSCZ) and first-episode schizophrenic (FESCZ) patients recruited from inpatients at five clinical centers in the Shaanxi Province, China, including 1) Department of Psychiatry of TFAHXJTU and Mental Health Center of 2) Baoji City, 3) Xianyang City, 4) Weinan City, and 5) Hanzhong City. Diagnoses were according to the Diagnostic and Statistical Manual of Mental Disorders, fourth Edition (DSM-IV). The current episode of ARSCZ patients was required to happen in the last three months. ARSCZ patients were free of treatment for at least six months. The current episode of FESCZ patients was required to happen in the last year. FESCZ patients did not take any antipsychotics or their accumulative dosages of antipsychotics intake were much less than effective dosage (generally less than 100 mg chlorpromazine equivalents in the last two weeks based on the transformation method described by ref. ^1^). Healthy controls (HC) were recruited from native residents of communities and villages in same the city where the clinical center was located. Healthy controls were not diagnosed with any mental disorders and were selected to be well-matched to the patients in relation to demographic features, socio-economic levels, alcohol and tobacco use, and diet habit. Generally, participants were excluded if they: 1) had current physical illness (such as diabetes, heart disease, thyroid disease, autoimmune disease or any recent infections) or DSM-IV axis I or axis II disorders (except schizophrenia in patients); 2) were taking other prescribed medications that could affect the central nervous, endocrine, or immune system; 3) were pregnant; 4) had acute digestive tract disorder during the last 30 days; and 5) had taken any antibiotics during the last 30 days. Two professional psychiatrists interviewed all participants and evaluated their mental status, using The Structured Clinical Interview for DSM-IV (SCID) and/or Mini-International Neuropsychiatric Interview. Other physical illnesses were evaluated by a physician through interview, physical examination, blood, and imaging. The discovery cohort included 171 (90 cases and 81 controls) subjects, and sampling took place at the five centers during March 2016 to October 2016. The validation cohort included 90 subjects (45 cases and 45 controls), and sampling took place in 2018 at the five centers. A total of 38 schizophrenic patients in the discovery cohort were followed up after three-month treatment. Antipsychotics-induced metabolic problems were assessed via measuring blood glucose and lipid, body weight, and abdomen circumference. All interviews were carried out in the morning immediately after the fasting blood and feces samples were collected.

Demographic information of participants is shown in Supplementary Data 1 and Supplementary Data 9.

**Symptom and cognitive assessment**

All assessments were conducted independently by two psychiatrists on the day when the blood samples were collected. These two psychiatrists underwent a training session to improve their assessment skills before the study to maintain a correlation coefficient greater than 0.8 for inter-rater reliability on the total score of each assessment. Clinical psychopathological symptoms were evaluated by the Positive and Negative Syndrome Scale (PANSS) (Supplementary Data 2)^2^. Cognitive functioning was assessed via the MATRICS Consensus Cognitive Battery (MCCB)^3,4^. These measurements have been used in the Chinese populations and show good validity and reliability^5,6^. The MCCB includes nine tests that assess seven cognitive domains: (1) Speed of processing: Trail Making Test, part A (TMT); Brief Assessment of Cognition in Schizophrenia: Symbol Coding (BACS SC); Category Fluency: Animal Naming (Fluency); (2) Attention and vigilance: Continuous Performance Test-Identical Pairs (CPT-IP); (3) Working memory: Wechsler Memory Scale-Third Edition (WMS-III), Spatial Span (SS) test; (4) Verbal learning: Hopkins Verbal Learning Test-Revised (HVLT-R); (5) Visual learning: Brief Visuospatial Memory Test-Revised (BVMT-R); (6) Reasoning and problem solving: Neuropsychological Assessment Battery (NAB): Mazes; (7) Social cognition: Mayer-Salovey-Caruso Emotional Intelligence Test (MSCEIT^TM^), and managing emotions subtest. The participants who were not recruited in Xi’an were unable to complete the CPT-IP test, as it needs to be done on a desktop computer in TFAHXJTU. Thus, this test was not included in our analysis. The methods for transformation of raw data of cognitive function were performed according to those used in the studies of Chuan Shi^6^ (Supplementary Data 3).

**Building a bacterial classifier using random forest.**

Five-fold cross-validation was performed ten times on a random forest model using the mOTUs abundance profiles of the schizophrenic patients and HCs (R package randomForest 4.6-14 ). The test error curves from ten trials of five-fold cross-validation were averaged. We chose the model which minimized the sum of the test error and its standard deviation in the averaged curve^7^. The probability of schizophrenia was calculated using this set of mOTUs and a receiver operating characteristic (ROC) was drawn (R 3.3.2, pROC package).

**Behavioral testing**

All behavioral tests were conducted between 8:30 AM and 04:00 PM and were analyzed using a video-computerized tracking system (SMART 3.0; Panlab SL, Barcelona, Spain) or were scored by manual observation. Previous experiments analyzing the influences of gut microbiota manipulations on the relevant behavioral tasks determined that sufficient results were achieved when group sizes were at least 7~9 animals^8,9^. We used 15~16 mice per group in behavioral tests and no statistical methods were used to predetermine the sample size. The investigators were blinded to allocation during experiments and outcome assessment. Generally, two experienced observers separately evaluated the behaviors of the mice and average scores were used for statistical analysis. Animals were given 30-minutes of habituation in the behavioral testing room. Tests were performed from the least to the most invasive with at least 48-hour intervals to minimize the influence of prior test history^10^. The behavioral tests were carried out in the following order: open field test (OFT), elevated plus maze (EPM), three-chamber social test (TCST), Barnes maze (BM), and tail suspension test (TST).

**Open-field test:** Mice were placed in an open-field arena (45 × 45× 45 cm) and allowed to explore freely for 30 minutes. The distance travelled and the time spent in the central zone (15× 15 cm squared arena) were analyzed. The incidence of rearing activity and jumping was recorded by manual observation.

**Elevated plus-maze test:** Mice were placed in the central square (10 cm x 10 cm) of a plus-shaped maze with two open and two closed arms (30 cm long, 10 cm wide, 20 cm walls, and 1 m above the ground). The time spent in each of the arms was manually recorded using filmed video. Entry in an arm required the animal to enter that arm with all four paws. The total time spent in the open arms and the ratio open/closed arms time were used as anxiety measures since anti-anxiolytic drugs decrease such quantities.

**Three-chamber sociability test:** The testing apparatus was a stainless steel box with three chambers (60 cm x 40 cm x 23 cm). The dividing chamber walls possessed openings to allow mice to access each chamber. During the habituation phase, the test mouse was placed in the center chamber—without access to side chambers—and allowed to freely explore for 10 mins. Following this, an unfamiliar sex- and strain-matched conspecific (stranger) was placed within a round, wire cup in one of the side chambers (social chamber). An identical inverted wire cup containing a novel object was placed in the other side chamber (non-social chamber). During the sociability phase, the test mouse was placed in the center chamber and allowed to freely explore all three chambers for 10 minutes. Distanced moved and time spent in each chamber were recorded by a video camera.

**Barnes maze test:** The protocol of the Barnes maze has been described previously^11^. Briefly, the circular maze consisted of 20 small holes, each with a diameter of 5 cm, and was placed 40 cm above the ground. The escape cage was made of PVC that was similar in color and material to the maze. The maze was placed in the center of a dedicated room and four pieces of paper in different colors and shapes were installed around the room as visual information. After each mouse was tested, the maze quadrant and the entire maze were cleaned with 70 percent alcohol. After each round of training, the maze was rotated clockwise to avoid interfering with smell or visual cues.

The Barnes maze test was divided into three stages: adaptation period (1 day), training period (3 days, trained twice on the first day, twice on the second day and once on the third day, respectively), and probe (1 day). There were three indicators recorded at the training stage (Primary latency, Latency, and Primary holes searched). Primary latency was defined as the time when the mice first found the target hole in 2 minutes, but not every time they entered the escape cage. Latency was defined as the time taken for the mice to find the target hole for the first time in 2 minutes and not enter it, but to continue to explore other holes. The time limit, however, did not exceed 2 minutes. Primary HS was defined as the number of times that the mice used its nose or head to explore other holes during the first exploration of the target hole within 2 minutes (to record once, the mice need to use their nose or head to explore the same hole, and would stand in the same position and keep exploring with their nose or head). During probe, the time spent in each quadrant and the HS of each quadrant (the number of times the mice bypassed each hole with their nose or head) were recorded during the test period. All sessions were recorded using Video and Smart software.

**Tail suspension test:** This test was carried out according to previous methods^12^. The part of the mouse tail almost 1.5 cm far from the tip was attached to a paper clip. The connection between clip and tail was fixed by medical tape. Mice were tested individually for six minutes, which was recorded by video camera. The immobility was defined as mice was hanging passively and without any movement. Any mice that climbed their tail were excluded from subsequent data analysis. Number of immobility episodes and total duration of immobility were recorded manually by a trained treatment blinded observer watching the videos.

**Specimen collection and preparation**

Peripheral venous blood was collected from patients and controls by venipuncture into vacutainer blood collection tubes between 7:00 AM and 9:00 AM. Serum was immediately isolated via centrifugation at 3000 g for 10-minutes. During the mouse experiment, fecal pellets were collected at several time points (Supplementary Fig. 4) and immediately stored in liquid nitrogen. DNA of the fecal sample was extracted using Qiagen QIAamp DNA Stool Mini Kit (Qiagen) according to the manufacturer’s instructions. After behavioral test, mice were euthanatized, small intestinal tissues, cecal tissues, colonic tissues, colonic contents, peripheral blood leukocyte, serum, brain tissues, were harvested and stored at -80 °C. Colon samples and the contents in colon were homogenized in ice-cold PBS. The supernatants were collected and stored at -80 °C for ELISA.

**Quantification of bacterial DNA in the feces of mouse**

Bacterial load in the feces of mice along transplantation was measured according to the methods reported in our previous study^13^. The fecal DNA mass was quantified using Quant-iT Pico-Green dsDNA Assay kit (Invitrogen) following the manufacturer’s instructions. Gene copy numbers were measured via qPCR and the sequence of a pair of primers targeting a universal and conservative fragment of 16S rRNA gene is showed in Supplementary Data. 15. A plasmid DNA including corresponding conservative sequence of 16S rRNA gene of *Escherichia coli* was loaded at a series of various concentrations (gradient dilution: 35.516 nM、7.1032 nM、1.42064 nM、0.284128 nM、0.0568256 nM、0.01136512 nM,). These 6 molar concentrations of plasmid DNA and their Ct values assayed by qPCR was used to construct linear regression equation (R^2^ > 0.98). The absolute copy number of 16S rRNA gene of each sample was calculated via putting its Ct value into the regression equation. Bacterial copy number in a fecal sample was normalized by its total DNA mass.

**Ultra-high-performance liquid chromatography-tandem mass-spectrometry (UHPLC-MS/MS) Assay**

Tissue concentrations of dopamine (DA), serotonin (5-HT), 4-aminobutyric acid (GABA), and tryptophan were analyzed in samples of the prefrontal cortex (PFC), the striatum, and the hippocampus (Supplementary Data 13). After the addition of 200 μL of extraction solution (precooled at -20 °C, acetonitrile-methanol-water, 2:2:1), the samples were vortexed for 30 seconds, and sonicated for 5 minutes in an ice-water bath. The vortex and sonication cycles were repeated 3 times, followed by incubation at -20 °C for 1 hour and centrifugation at 12000 rpm and 4 °C for 15 minutes. An 80 μL aliquot of the clear supernatant was transferred to an auto-sampler vial for analysis. Stock solutions were individually prepared by dissolving or diluting each standard substance to give a final concentration of 10 mmol/L. An aliquot of each of the stock solutions was transferred to a 10 mL flask to form a mixed working standard solution. A series of calibration standard solutions were then prepared by stepwise dilution of this mixed standard solution. The UHPLC separation was carried out using an Agilent 1290 Infinity II series UHPLC System (Agilent Technologies), equipped with an Agilent ZORBAX Hilic Plus column (50×2.1 mm, 1.8 μm). The mobile phase A was 1% formic acid in water, and the mobile phase B was acetonitrile. The assay parameter for UHPLC-MS/MS assay are shown in Supplementary Data 15 in the online materials. The column temperature was set at 35 °C. The auto-sampler temperature was set at 4 °C and the injection volume was 1 μL.

**RNA-seq and bioinformatics analysis**

The dissected tissues were homogenized and dissolved in TRIzol (TRIzol, Thermo Fisher Scientific). The concentration and quality of the RNA was assessed by Nano Drop ND-1000 spectrophotometry (Nano Drop Technologies, USA). RNA integrity was evaluated using the Agilent 2100 Bioanalyzer (Agilent Technologies, USA) and the samples with a greater than 7.0 RIN were used for further assays. RNA-seq libraries were generated using the TruSeq v2 RNA sample prep (Illumina, San Diego, CA, USA); RNA-seq was performed using an Illumina HiSeq 2500 (Illumina, Santiago, CA, USA), at Shanghai Genergy Co., Ltd. (Shanghai, China). Paired-end reads were aligned to the mouse transcriptome with the STAR software^14^ and were assembled into transcripts using the StringTie software^15^. Gene expression levels were presented as Fragments Per Kilobase of transcript per Million fragments mapped (FPKM). The gene-level FPKM values were then normalized using the log2 values (RPKM + 1) for further analyses. Differential expression was determined with DESeq2 software. The Benjamini-Hochberg correction method was used to control the false-discovery rate (FDR). The genes with fold change ≥ 2.0 and *P* value less than 0.05 were considered as differentially expressed. All significant genes were annotated for enriched biological functions and pathways using gene ontology (GO) enrichment analysis and Kyoto Encyclopedia of Genes and Genomes (KEGG) terms. GO was performed with KOBAS2.0 software^16^. GO provides label classification of gene function and gene product attributes (http://www.geneontology.org). GO analysis covers three domains: cellular component (CC), molecular function (MF), and biological process (BP)^17^. Significant canonical pathways had adjusted *P* values, according to Benjamini’s method, to be below 0.05.

**Supplementary Figure 1~9**

**
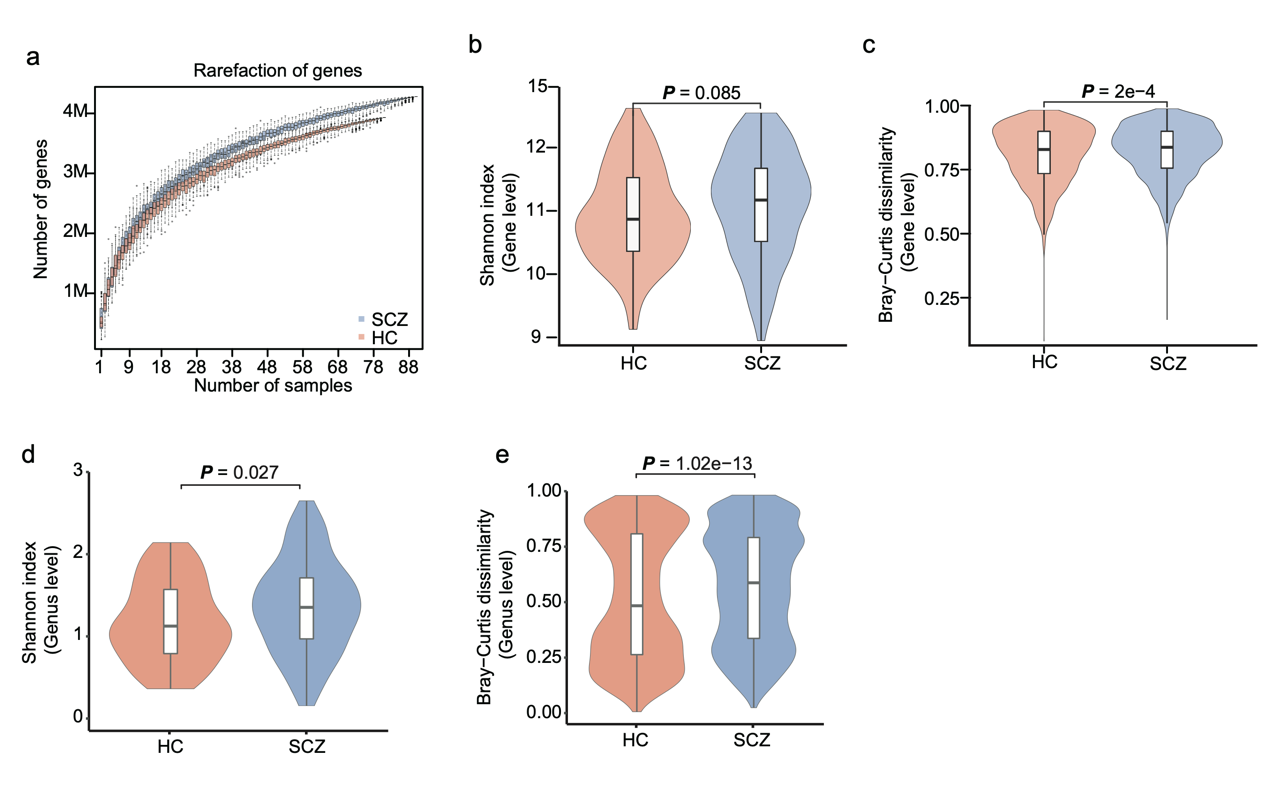
**

**Supplementary Figure 1. Richness and diversity of the gut microbiome in SCZ.**

Comparison of fecal shotgun sequencing data of HCs (n = 81) and SCZ patients (n = 90). (a) Rarefaction curves based on the number of genes counted in HCs and 90 SCZ patients. (b, d) α-diversity (Shannon index; b: gene level, *P* = 0.085; d: genus level, *P* = 0.027; two-sided Wilcoxon rank sum test) ;(c, e) β-diversity (Bray-Curtis dissimilarity index; c: gene level, *P* = 2e-4; e: genus level, *P* = 1.02e-13; two-sided Wilcoxon rank sum test). Boxes represent the median and interquartile ranges (IQRs) between the first and third quartiles; whiskers represent the lowest or highest values within 1.5 times IQR from the first or third quartiles. Source data are provided as a Source Data file.


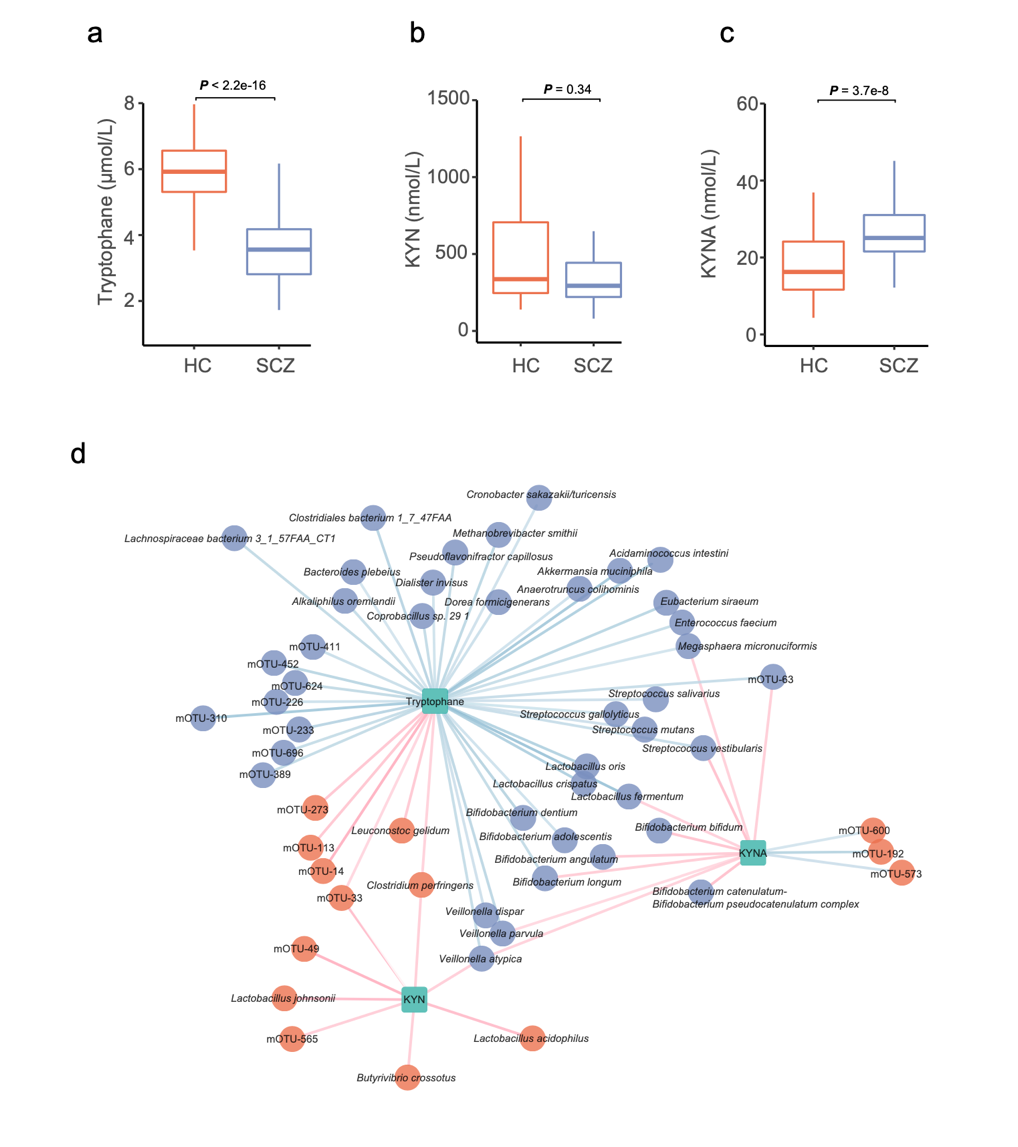


**Supplementary Figure 2. The alteration of tryptophan, kynurenine, and kynurenic acid and correlation with species that differ in abundance between healthy individuals and schizophrenic patients.**

The differences in the concentrations of serum tryptophan (a, two-sided Wilcoxon rank sum test), serum kynurenine (KYN) (b, two-sided Wilcoxon rank sum test), and kynurenic acid (KYNA) (c, two-sided Wilcoxon rank sum test) between healthy controls (HCs) (n = 81) and schizophrenic (SCZ) patients (n = 90); (d) The correlation (spearman correlation, two-sided *P* < 0.05) between tryptophan, KYN, and KYNA and significantly enriched species (*P* < 0.05, Wilcoxon rank sum test). Boxes represent the median and interquartile ranges (IQRs) between the first and third quartiles; whiskers represent the lowest or highest values within 1.5 times IQR from the first or third quartiles (a-c). Blue circles, species enriched in SCZ patients; red circles, species enriched in HCs; green squares, neurotransmitters. Blue edges, negative correlations; red edges, positive correlations. The gradation of color of the edges decreases with the absolute value of the Spearman’s (d).Source data are provided as a Source Data file.


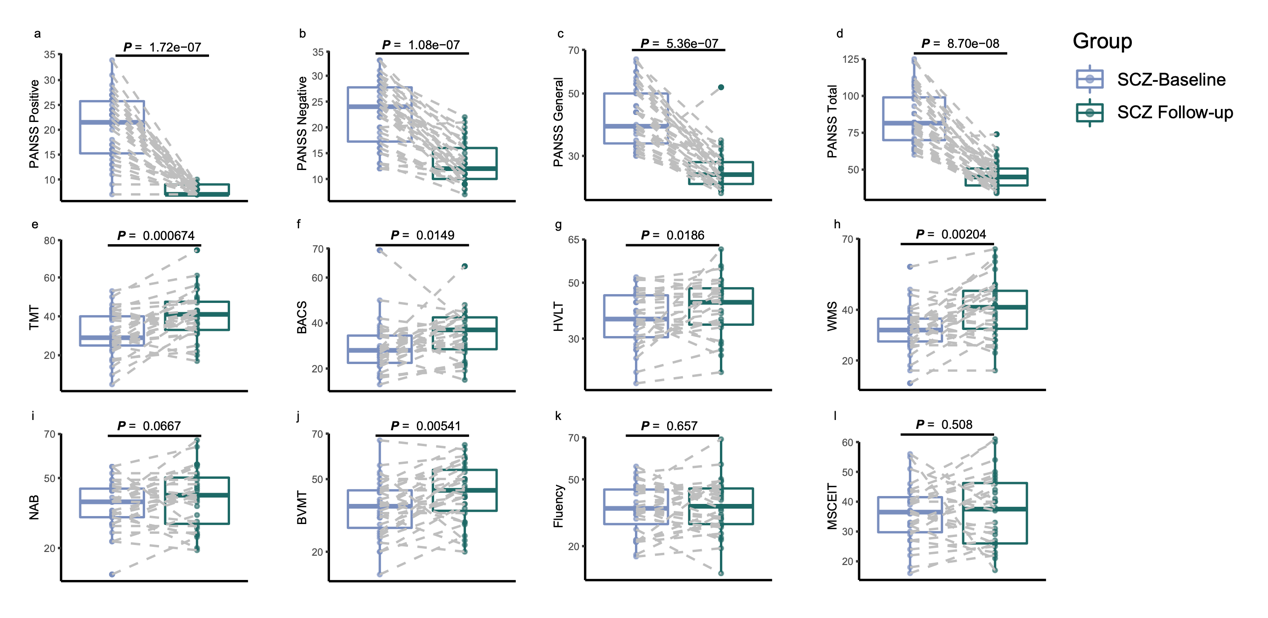


**Supplementary Figure 3. Symptom remission after treatment with antipsychotics.**

PANSS positive score (a, *P*=1.72e-07, n = 38), PANSS negative score (b, *P*=1.08e-07, n =38), PANSS general score (c, *P* = 5.36e-07, n =38) and PANSS total score (d, *P* = 8.70e-08, n = 38) before and after treatment. MCCB TMT (e, *P* = 0.000674, n = 31), BACS SC (f, *P* = 0.0149, n =31), HVLT (g, *P* = 0.0186, n =31), WMS (h, *P* = 0.00204, n =31), NAB (I, *P* = 0.0667, n =31), BVMT (j, *P* = 0.00541, n =31), Fluency (k, *P* = 0.657, n =31), MSCEIT (l, *P* = 0.508, n =28) before and after treatment. (Paired Wilcoxon rank sum test, two-sided) The dot represents one value from individual participants and Boxes represent the median and interquartile ranges (IQRs) between the first and third quartiles; whiskers represent the lowest or highest values within 1.5 times IQR from the first or third quartiles. Outliers are not shown. The line between dots represents the same participant. Source data are provided as a Source Data file.

**Supplementary Figure 4. Schematic diagram of bacterial transplantation and behavioral test.**

The gut microbiota of mice was depleted by antibiotics, and then either the storage buffer (saline), or *Streptococcus vestibularis* (*S. vestibularis*), or *Streptococcus thermophilus* (*S. thermophilus*) was administered via oral gavage and drinking water. A series of behavioral tests were carried out after administration of bacteria. OFT open field test, TCST three-chamber social test, EPM elevated plus maze, BM Barnes maze, and the TST tail suspension test (Detailed version related to Fig. 4a).

**Supplementary Figure 5. The changes in the abundance of *Streptococcus vestibularis* and *Streptococcus thermophilus* after bacterial transplantation.**

DNA abundance of the 16S rRNA gene of *Streptococcus vestibularis* or *Streptococcus thermophilus* was determined in feces from the mice colonized with *Streptococcus vestibularis*, *Streptococcus thermophilus* and saline-treated mice by q-PCR (n = 15 mice/treatment group/two independent experiments). Transplantation of *Streptococcus vestibularis* (a) or *Streptococcus thermophilus* (b) by oral gavage resulted in a significant increase in the concentration in feces, both on the day immediately after transplantation (*P* < 0.0001) and the day after the behavioral test (*P* < 0.0001). Data are representative of two independent experiments and presented as Means ± SEM. The circle represents one value measured in the feces of individual mice. *P* values were determined by one-way analysis of variance (ANOVA). See detailed statistical data in supplementary Source Data file.

**Supplementary Figure 6. Transplantation of *Streptococcus vestibularis* *(S. vestibularis*) or *Streptococcus thermophilus* *(S. thermophilus)* had no effects on the recipients’ learning and memory, anxious state, and depressive behavior compared to saline-treated mice.**

(a) Primary latency to find an escape hole in a 120-second training task of Barnes maze (BM) along with 5 trials in 3 days. There was no significant impact of bacteria on spatial memory acquisition (Group effect: *P* > 0.05; Time effect: *P* < 0.0001; repeated measure two-way analysis of variance (ANOVA) followed by Sidak's multiple comparisons test). Blue *P* value indicates the comparison between *S. V.*-treated and saline-treated mice and green *P* value indicates the comparison between *S. T.*-treated and saline-treated mice. (b) Time spent in the four quadrants by three groups of mice during 2-minute probe test of BM. Chance amount of time spent exploring each quadrant is 30-seconds out of 120-seconds. The three groups of mice have preference for the target quadrant (vs other quadrants), while there was no significant difference in the time spent in probing the target quadrant. n = 16, 15, 15 mice with oral gavage of saline, *S. V.*, and *S. T.*, respectively, in one BM test. (c) Time in open arm of elevated plus maze during 5-min exploration did not vary between any two groups. n = 13 mice/treatment group/an independent test. (d) Immobility time in tail suspension test was similar between any two groups. n = 11, 10, 10 mice with oral gavage of saline, *S. V.*, and *S. T.*, respectively, in one test. *P* values were determined by one-way ANOVA in b, c, d. The data are representative of two independent experiments and are presented as Means ± SEM. The circle represents one value from individual mice (b~d). See detailed statistical data in supplementary Source Data file.

**Supplementary Figure 7. Transplantation of *Streptococcus vestibularis* or** ***Streptococcus thermophilus* did not induce obvious weight loss, systemic inflammation, alterations in serum corticosterone and histological changes in main organs between the treatment groups.**

(a) The changes in weight in the three experimental groups, *Streptococcus vestibularis*-treated mice, *Streptococcus thermophilus-*treated mice, and saline-treated mice. There were no significantly impact of bacteria on body weight (Group effect: *P* > 0.05; Time effect: *P* < 0.0001; repeated measure two-way analysis of variance (ANOVA) followed by Sidak's multiple comparisons test). n = 17, 16, 17 mice with oral gavage of saline, *S. V.*, and *S. T.*, respectively, in one experiment. Pro-inflammatory cytokines, including IFN-γ (b), TNF-α (c), and IL-1β (d) in serum did not vary between the three groups of mice (*P* = 0.685, 0.739, 0.950, respectively). Serum corticosterone (e, *P* = 0.384), serum ACTH (f, *P* = 0.203), serum noradrenaline (g, *P* = 0.811), serum adrenaline (h, *P* = 0.336) and serum LPS (I, *P* = 0.578) did not vary between the three groups of mice. *P* values were determined by one-way ANOVA (b-j). Means ± standard error of the mean are presented; n = 9, 9, 8 mice with oral gavage of saline, *S. V.*, and *S. T.*, respectively, in an assay of serum cytokines and corticosterone (b-e). n = 8 mice/treatment group/one assay of serum ACTH, noradrenaline, adrenaline, and LPS (f-i). The circle represents one value from individual mice (b-i). (j) The transplantation did not cause obvious pathological changes of major organs in saline-treated mice (top) or *S. vestibularis*-treated mice (bottom). The organs were stained with hematoxylin and eosin (HE). HE staining was performed using tissues from three mice per group in two independent experiments. We obtained similar results in the two experiments and representative figures were selected from one experiment. IFN: interferon; TNF: tumor necrosis factor; IL: interleukin; ACTH: adrenocorticotropic hormone. See detailed statistical data in supplementary Source Data file.

**Supplementary Figure 8. The presence of *Streptococcus vestibularis* in the mouse gut modulates neurotransmitter concentration in serum, proximal colon, and intestinal content.**

(a, b, c) Levels of 5-hydroxytryptamine (5-HT), serum dopamine (DA), and 4-aminobutyric acid (GABA) in mice receiving either *Streptococcus vestibularis* (*S. vestibularis*) or saline on day 25 (at the end of the transplantation) and 35 (after behavioral tests). (d, e, f) Levels of 5-HT, DA, and GABA on day 25 and 35 in the intestinal contents of mice colonized with either *S. vestibularis* or saline. (g, h, i) Levels of 5-HT, DA, and GABA on day 25 and 35 in colonic tissue of mice colonized with *S. vestibularis* or saline. n = 6 mice/treatment group/one assay. Data are expressed as mean ± standard error of the mean, statistics are calculated by two-way analysis of variance followed by post hoc Sidak's test (time factor: at the end of FMT vs. 10 days after FMT; microbiota factor: bacterium vs. saline). *S. vestibularis* colonization decreased DA in serum (*P* = 0.026), intestinal contents (*P* = 0.017) and colonic tissue (*P* = 0.004) on day 25, decreased GABA in intestinal contents on day 25 (*P* = 0.003) and increased 5-HT in intestinal contents on day 35 (*P* = 0.028). See detailed statistical data in supplementary Source Data file.

**Supplementary Figure 9. Functional annotations of differently expressed genes in the gut and the brain of *Streptococcus vestibularis*-treated mice via GO enrichment analysis and KEGG pathways analysis.**

The top 20 KEGG pathways (left) and the top 10 pathways of biological processes (BP), cellular components (CC), and molecular functions (MF) in GO (right), which were significantly enriched by the differentially expressed genes (at least 2-fold and *P* value < 0.05) in the small intestine (a, b), the colon (c, d), the prefrontal cortex (e, f), the striatum (g, h), and the hippocampus (i, j) of *Streptococcus vestibularis-*treated mice. GO, Gene Ontology; KEGG, Kyoto Encyclopedia of Genes and Genomes. See detailed statistical data in supplementary Source Data file.

**Reference:**

1. Woods, S.W. Chlorpromazine equivalent doses for the newer atypical antipsychotics. *J Clin Psychiatry* **64**, 663-667 (2003).

2. Kay, S.R., Fiszbein, A. & Opler, L.A. The positive and negative syndrome scale (PANSS) for schizophrenia. *Schizophrenia bulletin* **13**, 261-276 (1987).

3. Kern, R.S.*, et al.* The MCCB impairment profile for schizophrenia outpatients: results from the MATRICS psychometric and standardization study. *Schizophrenia research* **126**, 124-131 (2011).

4. Nuechterlein, K.H.*, et al.* The MATRICS Consensus Cognitive Battery, part 1: test selection, reliability, and validity. *Am J Psychiatry* **165**, 203-213 (2008).

5. Wu, J.Q.*, et al.* Cognitive impairments in first-episode drug-naive and chronic medicated schizophrenia: MATRICS consensus cognitive battery in a Chinese Han population. *Psychiatry Res* **238**, 196-202 (2016).

6. Shi, C.*, et al.* The MATRICS Consensus Cognitive Battery (MCCB): Co-norming and standardization in China. *Schizophrenia research* **169**, 109-115 (2015).

7. Zeller, G.*, et al.* Potential of fecal microbiota for early-stage detection of colorectal cancer. *Molecular systems biology* **10**, 766 (2014).

8. Bruce-Keller, A.J.*, et al.* Obese-type gut microbiota induce neurobehavioral changes in the absence of obesity. *Biol Psychiatry* **77**, 607-615 (2015).

9. Buffington, S.A.*, et al.* Microbial Reconstitution Reverses Maternal Diet-Induced Social and Synaptic Deficits in Offspring. *Cell* **165**, 1762-1775 (2016).

10. McIlwain, K.L., Merriweather, M.Y., Yuva-Paylor, L.A. & Paylor, R. The use of behavioral test batteries: effects of training history. *Physiology & behavior* **73**, 705-717 (2001).

11. Attar, A.*, et al.* A shortened Barnes maze protocol reveals memory deficits at 4-months of age in the triple-transgenic mouse model of Alzheimer's disease. *PLoS One* **8**, e80355 (2013).

12. Bergner, C.L.*, et al.* Mouse models for studying depression-like states and antidepressant drugs. *Methods Mol Biol* **602**, 267-282 (2010).

13. Zhu, F.*, et al.* Transplantation of microbiota from drug-free patients with schizophrenia causes schizophrenia-like abnormal behaviors and dysregulated kynurenine metabolism in mice. *Molecular psychiatry* (2019).

14. Zhang, P., Hung, L.H., Lloyd, W. & Yeung, K.Y. Hot-starting software containers for STAR aligner. *Gigascience* **7**(2018).

15. Pertea, M.*, et al.* StringTie enables improved reconstruction of a transcriptome from RNA-seq reads. *Nat Biotechnol* **33**, 290-295 (2015).

16. Ai, C. & Kong, L. CGPS: A machine learning-based approach integrating multiple gene set analysis tools for better prioritization of biologically relevant pathways. *J Genet Genomics* **45**, 489-504 (2018).

17. He, K., Wang, Q., Yang, Y., Wang, M. & Pan, Y. A Comparative Study of Mouse Hepatic and Intestinal Gene Expression Profiles under PPARalpha Knockout by Gene Set Enrichment Analysis. *PPAR Res* **2011**, 629728 (2011).
