# Supplementary material for: Metagenome-wide association of gut microbiome features for schizophrenia
Source: Nat Commun. 2020 Mar 31;11:1612. doi: 10.1038/s41467-020-15457-9 (PMC7109134; doi:10.1038/s41467-020-15457-9)
Supplement: Supplementary file 3 — Description of Additional Supplementary Files [file 41467_2020_15457_MOESM3_ESM.pdf]

Description of Additional Supplementary Files

File Name: Supplementary Data 1
Description: Phenotypes of baseline volunteers. (Excel file)

File Name: Supplementary Data 2
Description: POSITIVE AND NEGATIVE SYNDROME SCALE (PANSS) for SCZ patients. (Excel file)

File Name: Supplementary Data 3
Description: Cognitive assessment of participants by MATRICS consensus cognitive battery (MCCB). (Excel file)

File Name: Supplementary Data 4
Description: Statistics for the metagenomic shot-gun sequencing data. (Excel file)

File Name: Supplementary Data 5
Description: a: Significantly differentially enriched mOTU between SCZ patients and healthy controls. (Excel file);

b: Significantly differentially enriched genus between SCZ patients and healthy controls. (Excel file);

File Name: Supplementary Data 6
Description: a: Kyoto Encyclopedia of Genes and Genomes (KEGG) functional modules differentially enriched in gut microbiota between SCZ patients and controls (Excel file).

b: Kyoto Encyclopedia of Genes and Genomes (KEGG) functional pathways differentially enriched in gut microbiota between SCZ patients and controls (Excel file).

File Name: Supplementary Data 7
Description: a: The presence of GBMs in 42 species (Excel file).

b: The pvalue of GBMs in 42 species in our healthy people and SCZ patients (Excel file).

File Name: Supplementary Data 8
Description: The effect of confounders in RF classifier (Excel file).

File Name: Supplementary Data 9
Description: a: Phenotypes of validation cohort (Excel file).

b: Statistics for the metagenomic shot-gun sequencing data of validation cohort (Excel file).

File Name: Supplementary Data 10
Description: Significantly differentially enriched mOTU between healthy controls and 38 baseline SCZ patients or 38 treated SCZ patients. (Excel file)

File Name: Supplementary Data 11
Description: Alterations in abundance of mOTUs in the gut of SCZ patients after 3-month antipsychotics treatment (Excel file).

File Name: Supplementary Data 12
Description: Expressed gene in the intestine(a), colon(b), PFC(c), striatum(d) and hippocampus(e) of the mice transplanted with Streptococcus vestibularis or saline(Excel file).

File Name: Supplementary Data 13
Description: Concentrations of dopamine, 5-hydroxytryptamine, 4-aminobutyric acid, and tryptophan in various brain regions of the mice transplanted with Streptococcus vestibularis or saline (Excel file).

File Name: Supplementary Data 14
Description: ELISA kit summary (Excel file).

File Name: Supplementary Data 15
Description: The primers of qPCR in this study (Excel file).
